# The Higher the CKD Stage, the Higher the Psychological Stress in Patients with CKD during COVID-19 Pandemic

**DOI:** 10.3390/jcm11164776

**Published:** 2022-08-16

**Authors:** Kyung-Mi Lee, Ji-Sun Kim, Sungjo Hwang, Nam Jun Cho, Samel Park, Hyo Wook Gil, Eun Young Lee

**Affiliations:** 1 Department of Internal Medicine, Soonchunhyang University Cheonan Hospital, Cheonan 31151, Korea; 2 Department of Psychiatry, Soonchunhyang University Cheonan Hospital, Cheonan 31151, Korea; 3 Institute of Tissue Regeneration, College of Medicine, Soonchunhyang University, Cheonan 31151, Korea; 4 BK21 Four Project, College of Medicine, Soonchunhyang University, Cheonan 31151, Korea

**Keywords:** COVID-19, CKD, psychological distress

## Abstract

The coronavirus disease 2019 (COVID-19) pandemic is related to psychological distress. Such distress depends on various factors. We previously reported that hemodialysis patients have more psychological distress than peritoneal dialysis patients among patients on dialysis in the COVID-19 pandemic era. However, no study has reported how psychological distress related to the COVID-19 pandemic depends on renal function in the entire group of chronic kidney disease (CKD) patients. Therefore, the objective of this study was to investigate psychological distress and concerns related to COVID-19 according to CKD stage. This was a cross-sectional study that included 397 CKD patients who visited a hospital from August 2020 to November 2020. Patients responded to questionnaires covering depression (9-item Patient Health Questionnaire, PHQ-9), anxiety (7-item Generalized Anxiety Disorder, GAD-7), psychological impact of event (22-item Impact of Event Scale-Revised, IES-R), insomnia (7-item Insomnia severity Index, ISI), concerns, and precautionary measures about COVID-19. According to eGFR and dialysis status, patients were divided into three groups: (1) patients with CKD stage 1~2, (2) patients with CKD stage 3~5 without dialysis, and (3) dialysis patients. The higher the CKD stage, the higher the GAD-7 (*p* = 0.009) and the ISI score (*p* = 0.001). When patients with CKD stage 1~2 and CKD stage 3~5 (with or without dialysis) were compared, PHQ-9 (*p* = 0.026), GAD-7 (*p* = 0.010), and ISI score (*p* = 0.002) were higher in the CKD stage 3~5 group. However, when comparing those with and without dialysis, only the ISI score (*p* = 0.008) showed a significant difference. More severe kidney dysfunction in CKD patients was associated with more psychological distress during the COVID-19 pandemic. Therefore, as CKD stage increases, more attention should be paid to the mental care of these patients.

## 1. Introduction

The outbreak of novel coronavirus, called severe acute respiratory syndrome coronavirus 2 (SARS-CoV-2), was initially reported to the World Health Organization (WHO) in December 2019. In March 2020, WHO declared that coronavirus disease 2019 (COVID-19) could be characterized as a pandemic, since this viral infection had spread rapidly to a number of countries [1]. As of 11 November 2020, the cumulative number of confirmed cases in Korea was 27,799, which was 441.1 per 100,000 population [2]. SARS-CoV-2 virus can affect the respiratory tract, gastrointestinal system, liver, heart, central nervous system, and kidney. It can lead to multiorgan failure [3]. Patients with older age and medical comorbidities are more likely to become infected, with worse outcomes [4].

The period of widespread occurrence of infectious diseases, such as COVID-19, may be closely related to psychological distress and symptoms of mental illness even in the general public without a psychiatric problem [5]. From the following sentence, psychological distress associated with the period of the COVID-19 pandemic will be referred to as “COVID-19-related psychological distress” for convenience. COVID-19-related psychological distress is caused not only by concerns about its infection, but also by large-scale isolation and overall socioeconomic problems [6]. Previous studies revealed the psychological impact of the period of the COVID-19 pandemic on anxiety, depressive symptoms, and sleep quality. Such an impact depends on age, gender, job status, specific physical symptoms (e.g., myalgia, dizziness, coryza), and preexisting psychiatric illness [7,8,9,10]. In addition, comorbidities, such as hypertension, diabetes mellitus, asthma, eczema, migraine, ischemic heart disease, and stroke, can affect mental health [8].

Likewise, one study suggested that older patients with chronic kidney disease (CKD) are more likely to develop psychiatric problems related to COVID-19 than other older patients without CKD [11]. A similar study was conducted on children with CKD [12]. As a result, children with CKD had more COVID-19-related anxiety than those without CKD [12]. Whether these results can be equally applied to general adults has not been confirmed. There are studies showing high levels of anxiety and depression in general adult-age patients receiving renal replacement therapy during the COVID-19 pandemic [13,14]. However, these studies did not include all CKD patients. They only included patients who underwent renal replacement therapy.

Other similar studies have reported the relationship between CKD and psychiatric problems before the COVID-19 pandemic. A study from western Rajasthan reported that 66% and 61% of CKD patients undergoing hemodialysis had depressive disorder and anxiety disorder, respectively [15]. One study observed that there is a direct linear correlation between CKD or ESRD and insomnia [16]. Specifically, a worsening CKD is directly associated with worsened insomnia [16]. It might be due to differences in comorbidities and individual perception of health caused by CKD. There are studies showing that the prevalence of inflammation and oxidant stress increases in CKD patients, leading to psychological distress, such as depression [17,18]. However, these studies were mainly conducted on dialysis patients. CKD patients not on dialysis, especially CKD stage 1~2 patients, were often excluded from these studies. We have previously reported psychological distress associated with COVID-19 in dialysis patients [19]. In that study, we compared hemodialysis (a procedure where a dialysis machine and a special filter called an artificial kidney, or a dialyzer, are used to clean one’s blood, performed 2–3 times a week at the hospital) patients and peritoneal dialysis (a treatment for kidney failure that uses the lining of the abdomen to filter one’s blood inside the body and can be practiced daily at home) patients and observed that hemodialysis patients had more psychological distress related to COVID-19. A limitation of that study was that there was no comparison with non-dialysis patients.

In order to overcome that limitation of the previous study, we added stage 1~5 non-dialysis CKD patients to dialysis patients in the previous study and tried to compare the degree of psychological distress according to their CKD stage. We hypothesized that the worse the patient’s renal function, the greater the psychological stress related to COVID-19. 

## 2. Methods

### 2.1. Subjects

This was a cross-sectional, observational study of patients who received outpatient treatment at Nephrology Department of Soonchunhyang University Hospital Cheonan. The study protocol was approved by the Institutional Review Board of Soonchunhyang University Cheonan Hospital (No. 2020-07-029). All patients provided written informed consent before enrollment.

A survey was conducted from 17 August 2020 to 28 November 2020 when the monthly number of COVID-19 diagnosis cases had increased in Korea. A total of 397 patients with CKD defined by 2012 Kidney Disease: Improving Global Outcomes (KDIGO) guidelines who visited the Nephrology Department at Soonchunhyang University Cheonan were selected. Among patients selected for this study, patients with dialysis were included in the previous study [19]. Dialysis patients were taken part from August 2020 to September 2020. Although there is a slight difference in the timing, this period from August 2020 to November 2020 is when the number of confirmed COVID-19 patients was continuously maintained between 20,000 and 30,000, with little difference in incidence according to time.

Among them, those aged under 18 or above 90 years were excluded. Patients who could not complete the questionnaire due to cognitive degradation or illiteracy were excluded. Patients with any acute illness, hospitalization within three months, or a history of COVID-19 infection were also excluded.

Subjects were divided into three groups. First, according to Kidney Disease Outcomes Quality Initiative (KDOQI) definitions, non-dialysis patients were divided into two groups: stage 1–2 CKD patients with eGFR ≥ 60 mL/min and stage 3–5 CKD patients with eGFR < 60 mL/min according to eGFR calculated by Chronic Kidney Disease Epidemiology Collaboration (CKD-EPI) equation. Patients with dialysis, including both peritoneal dialysis and hemodialysis, were used as the third group.

### 2.2. Survey Development

We investigated the medical history and laboratory data of patients at the time the questionnaires were administered by reviewing medical records. Their medical records included age and sex, diabetes mellitus or hypertension, statin intake, visit interval, and laboratory data.

To measure psychological distress, the same questionnaire as in the prior study was used [19]. The 9-item Patient Health Questionnaire (PHQ-9) [20] indicating the degree of depression, the 7-item Generalized Anxiety Disorder scale (GAD-7) [21] indicating the degree of anxiety, the 22-item Impact of Event Scale-Revised (IES-R) [22] indicating the degree of psychological impact of events, and the 7-item Insomnia severity Index (ISI) [23] indicating the degree of insomnia were used. Each questionnaire was scored. The severity was divided into normal, mild, moderate, and severe according to each score (PHQ-9: 0–8, 9–14, 15–19, 20–27; GAD-7: 0–4, 5–9, 10–14, 15–21; IES-R: 0–8, 9–25, 26–43, 44–88; and ISI: 0–7, 8–14, 15–21, 22–28). 

To evaluate psychological distress related to COVID-19, a questionnaire was conducted on concerns and precautionary measures related to COVID-19 [8,19]. Concerns about COVID-19 included four questions. Precautionary measures related to COVID-19 included six questions. To determine whether time spent at home was related to psychological distress in CKD patients, correlation between each questionnaire score and time spent at home was analyzed.

### 2.3. Statistical Analysis

Jonckheere–Terpstra test [24] was used to confirm the tendency of demographic and psychological characteristics for patients with stage 1~2 CKD, patients with stage 3~5 CKD, and dialysis patients. Linear-by-linear association was used for categorical data. Student’s t-test was used to compare psychological characteristics between two groups (dialysis patients vs. non-dialysis CKD patients, CKD stage 3~5 patients (with or without dialysis) vs. CKD stage 1~2 patients). Logistic regression was utilized to exclude confounding factors such as HTN, DM, age, and sex. All statistical analyses were performed using SPSS version 26 (SPSS, Inc., Chicago, IL, USA). The significance level was set at *p*-value < 0.05. 

## 3. Results

### 3.1. Patient Characteristics

We included a total of 397 CKD patients who visited Soonchunhyang University Cheonan Hospital from 17 August 2020 to 28 November 2020 during the COVID-19 pandemic. These patients were divided into three groups: patients with stage 1~2 CKD (eGFR ≥ 60mL/min, *n* = 111), non-dialytic patients with stage 3~5 CKD (eGFR < 60 mL/min, *n* = 138), and patients on dialysis (*n* = 148).

Baseline characteristics of study participants are presented in Table 1. The mean age of patients was 53.5 years for patients on dialysis, 60.3 years for patients with stage 3~5 CKD, and 51.2 years for patients with stage 1~2 CKD (*p* = 0.199). Gender ratio in patients on dialysis was similar to that in patients with stage 3~5 CKD and patients with stage 1~2 CKD (*p* = 0.366). The median visit interval became shorter as renal function deteriorated (15.0 days for patients on dialysis, 79.0 days for patients with stage 3~5 CKD, and 127.3 days for patients with stage 1~2 CKD, *p* < 0.001). 

The poorer the kidney function, the higher the prevalence of diabetes mellitus (DM) and hypertension (HTN). In total, 141 (35.25%) patients were diagnosed and treated with DM, including 55 (37.26%) in the group of dialysis patients, 63 (45.65%) in the group of patients with stage 3~5 CKD, and 23 (20.72%) in the group of patients with stage 1~2 CKD (*p* = 0.014). A total of 181 (71.83%) patients had HTN, including 124 (83.78%) in the group of dialysis patients, 115 (83.33%) in the group of patients with stage 3~5 CKD, and 65 (58.56%) in the group of patients with stage 1~2 CKD (*p* < 0.001).

Laboratory data were compared among the three CKD groups. Hemoglobin and hematocrit tended to lessen as kidney function deteriorated. Hb and Hct were around 14.07 g/dL and 41.63% in patients with stage 1~2 CKD, 11.87 g/dL and 35.50% in patients with stage 3~5 CKD, and 10.61 g/dL and 31.06% in dialysis patients, respectively. Potassium tended to be increased as kidney function deteriorated (4.33 mmol/L in patients with stage 1~2 CKD vs. 4.62 mmol/L in patients with stage 3~5 CKD vs. 4.54 mmol/L in dialysis patients, *p* < 0.001). Albumin tended to be less as kidney function deteriorated (4.49 g/dL in patients with stage 1~2 CKD vs. 4.24 g/dL in patients with stage 3~5 CKD vs. 3.94 g/dL in dialysis patients, *p* < 0.001). However, cholesterol and statin intake according to renal function showed no trend. 

### 3.2. Psychological Measurements

**Figure 1:**
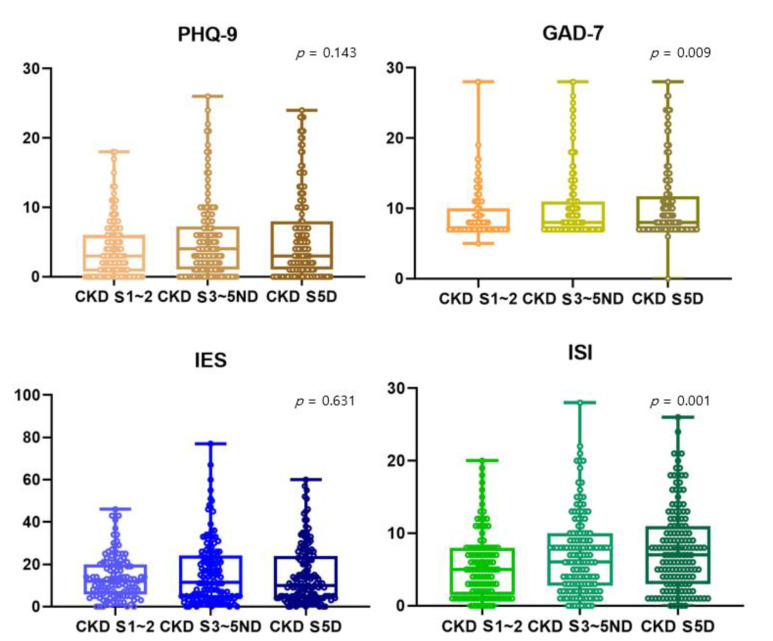
Comparison of scores of PHQ-9, GAD-7, IES-R, and ISI among stage 1~2 CKD patients, stage 3~5 non-dialytic CKD patients, and dialysis patients. The Jonckheere–Terpstra test was used to see if there was a statistical significance for the tendency. The higher the CKD stage, the higher the anxiety (9.00 ± 3.69 vs. 10.21 ± 4.87 vs. 10.51 ± 5.19, *p*-value = 0.009) and insomnia (5.62 ± 4.58 vs. 7.01 ± 5.45 vs. 7.88 ± 5.74, *p*-value = 0.001) scores. CKD S5D, chronic kidney disease stage 5 dialysis; S3~5ND, stage 3~stage 5 non-dialysis; S1~2, stage 1~stage 2; PHQ-9, the 9-item Patient Health Questionnaire; GAD-7, the 7-item Generalized Anxiety Disorder scale; IES-R, the 22-item impact of Event Scale-Revised; ISI, insomnia severity index.

In Figure 1, all scores reflecting depression (PHQ-9), anxiety (GAD-7), stress (IES-R), and insomnia (ISI) tended to be higher as kidney function deteriorated. However, PHQ-9 and IES-R showed no statistically significant trend. GAD-7 score reflecting anxiety was around 10.51 for dialysis patients, 10.21 for patients with stage 3~5 CKD, and 9.00 for patients with stage 1~2 CKD (*p* = 0.009). ISI score reflecting insomnia was around 7.88 for dialysis patients, 7.01 for patients with stage 3~5 CKD, and 5.62 for patients with stage 1~2 CKD (*p* = 0.001) (Figure 1).

**Figure 2:**
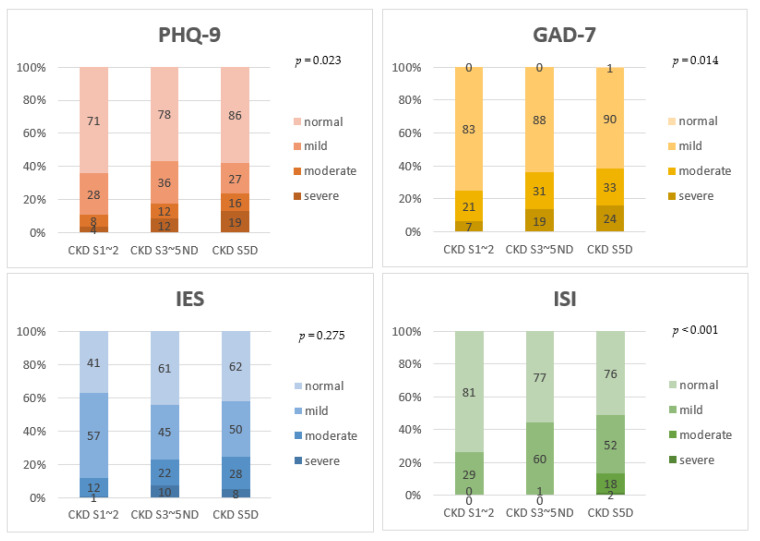
Comparison of severity of PHQ-9, GAD-7, IES-R, and ISI among stage 1~2 CKD patients, stage 3~5 non-dialytic CKD patients, and dialysis patients. The linear-by-linear association test was used to see if there was a statistical significance for the tendency. As CKD stage increased, the proportion of patients with severe depression, anxiety, and insomnia also increased. CKD S5D, chronic kidney disease stage 5 dialysis; S3~5ND, stage 3~stage 5 non-dialysis; S1~2, stage 1~stage 2; PHQ-9, the 9-item Patient Health Questionnaire; GAD-7, the 7-item Generalized Anxiety Disorder scale; IES-R, the 22-item impact of Event Scale-Revised; ISI, insomnia severity index.

In terms of severity, the proportion of patients who scored high on PHQ-9, GAD-7, and ISI showed a tendency to increase as the renal function worsened (Figure 2). About 10.8% of patients with stage 1~2 CKD, 17.4% of patients with stage 3~5 CKD, and 23.6% of dialysis patients reported moderate to severe depression (*p* = 0.023). About 25.2% of patients with stage 1~2 CKD, 36.3% of patients with stage 3~5 CKD, and 38.5% of dialysis patients reported moderate to severe anxiety (*p* = 0.014). About 26.4% of patients with stage 1~2 CKD, 44.2% of patients with stage 3~5 CKD, and 48.7% of dialysis patients reported insomnia. Among the entire CKD cohort, only two patients in the dialysis group had severe insomnia (*p* < 0.001).

Logistic regression was performed to exclude confounding factors, such as HTN, DM, age, and sex. The outcome was set as a score in PHQ-9, IES-R, and ISI, corresponding to mild to severe depression, psychological impact of events, and insomnia, respectively. Since the number of normal patients based on the GAD-7 score was too small (n = 1), patients with moderate to severe anxiety were analyzed for outcomes. As a result, there was no statistical association between confounding factors (such as HTN, DM, age, and sex) and psychological distress, except that male gender was 0.607-times (*p* = 0.027) less likely to have moderate to severe anxiety than female gender. However, there was a significant association between CKD stage and anxiety (*p* = 0.013). Compared to patients with stage 1~2 CKD, patients with stage 3~5 CKD without dialysis and dialysis patients were 2.119-times (*p* = 0.016) and 2.341-times (*p* = 0.004) more likely to have moderate to severe anxiety, respectively. There was a significant association between CKD stage and insomnia (*p* < 0.001). Compared to patients with stage 1~2 CKD, patients with stage 3~5 CKD without dialysis and dialysis patients were 2.535-times (*p* = 0.002) and 3.019-times (*p* < 0.001) more likely to have mild to severe insomnia, respectively (Table 2).

When patients with stage 3~5 CKD were grouped into one group based on eGFR, regardless of dialysis statues, and compared with stage 1~2 CKD patients, PHQ-9, GAD-7, IES-R, and ISI scores were higher in the group of patients with stage 3~5 CKD. There were statistically significant differences in PHQ-9, GAD-7, ISI score, and severity among patient groups. PHQ-9 score was around 5.40 for patients with stage 3~5 CKD and 4.01 for patients with stage 1~2 CKD (*p* = 0.026). GAD-7 score was around 10.37 for patients with stage 3~5 CKD and 9.00 for patients with stage 1~2 CKD (*p* = 0.010). ISI score was around 7.45 for patients with stage 3~5 CKD and 5.62 for patients with stage 1~2 CKD (*p* = 0.002) (Table 3).

Similarly, when patients with stage 1~5 CKD without dialysis were grouped into one group and compared with dialysis patients, all questionnaire scores were higher in dialysis patients. However, there was no statistically significant difference, except for ISI score and severity (Supplementary Table S1).

### 3.3. Concerns and Precautionary Measures about COVID-19 

Concerns and precautionary measures about COVID-19 in dialysis patients, stage 3~5 non-dialysis CKD patients, and stage 1~2 CKD patients are shown in Table 4. There was no statistically significant difference in concerns about COVID-19 in each group. Concerns about coming into contact with COVID-19 and concerns about the ability of doctors to diagnose were relatively low in all three groups. On the other hand, concerns about dying if infected with COVID-19 and concerns about other families were high.

Most patients answered that they followed precautions against COVID-19, especially covering mouth when coughing or sneezing and wearing a mask, regardless of the presence or absence of symptoms. There were no significant differences in precautionary measures for COVID-19 in each group, except for hours stayed at home per day to avoid COVID-19. Dialysis patients replied that they stayed at home for about 15.92 h per day, while stage 3~5 non-dialysis CKD patients and stage 1~2 CKD patients reported that they stayed at home for about 14.18 h per day and 13.00 h per day, respectively (*p* < 0.001).

As a result of the correlation study between each questionnaire score and the time spent at home, all questionnaire scores showed positive correlations with time stayed at home, although correlation coefficients were not large. Only the correlation between PHQ-9 score, suggesting depression, and time stayed at home showed statistical significance (*p* = 0.004) (Supplementary Figure S1).

When patients with stage 3~5 CKD, with or without dialysis, were compared with stage 1~2 CKD patients, results were similar to results when all three groups were compared (Table 5). However, when stage 1~5 CKD patients without dialysis were compared with dialysis patients, there was a difference in the perceived likelihood of contracting COVID-19 during the current outbreak. A larger percentage of non-dialysis CKD patients responded that they were likely to contract COVID-19 during the current outbreak (very likely: 1.6% vs. 0.7%; somewhat likely: 1.6% vs. 2.7%) (*p* = 0.042) (Supplementary Table S2).

## 4. Discussion

In this study, we investigated psychological stress, including depression, anxiety, psychological impact of events, and insomnia, in the era of the COVID-19 pandemic for the entire group of CKD patients. In the present study, it was observed that anxiety and insomnia worsened as CKD stage increased for all CKD patients, including those with CKD stage 1~2. The same result was confirmed even after adjusting other confounding factors, such as HTN, DM, age, and sex. In the case of depression, it showed no statistically significant difference between those without dialysis and those with dialysis. However, it was significantly different between those with stage 1~2 CKD (eGFR ≥ 60 mL/min) and those with stage 3~5 CKD (eGFR < 60 mL/min).

To see if psychological distress answered by patients through questionnaires in this study was related to COVID-19, we investigated COVID-19-related concerns and precautionary measurements. As a result, concerns about dying if infected with COVID-19 and concerns about other families were high. Precautionary measures of covering mouth when coughing or sneezing and wearing mask, regardless of the symptoms, were followed by most patients. There were no significant differences according to CKD stage. Therefore, anxiety and insomnia, which worsen as the CKD stage increases, are more likely due to differences in comorbidities, chronic inflammation, oxidant stress, and individual perception of health caused by CKD, rather than differences in responses due to COVID-19. In many cases, the prevalence of chronic inflammation and oxidant stress increases as the CKD stage increases, but since there are individual differences, it may also act as a confounding factor for COVID-19-related psychological distress [17,18].

The time spent at home increased as the CKD stage increased. Dialysis modality may affect the number of hours that patients spend at home. Of the 148 dialysis patients who participated in the study, 78 patients on hemodialysis needed to spend time away from home and 70 patients on peritoneal dialysis needed to stay at home in order to perform their treatments. The time spent at home had a correlation with PHQ-9 score. It is currently unclear whether CKD patients are staying home longer because they are depressed or whether they are depressed because they have been at home for longer due to their concerns about COVID-19.

Because subjects in this study were only CKD patients, we were unable to compare psychological stress or COVID-19-related concerns and precautionary measurements with people with normal renal function. In simple comparison with a study conducted on the general public in China’s COVID-19 epidemic area, several concerns were more prevalent in CKD patients, including concerns about other family members being diagnosed with COVID-19 infection (75.2% in general public vs. 86.1% in CKD patients) and unlikely to survive if infected with COVID-19 (30.8% in general public vs. 45.4% in CKD patients) [8]. However, since these comparisons did not consider differences according to the study area or demographic characteristics of subjects, additional comparative studies on patients with normal renal function are needed.

In addition to the above, this study has other limitations. First, we were unable to assess an individual’s psychological condition before the outbreak due to sudden occurrence of the COVID-19 outbreak. Since there were no data before COVID-19, we could not know how much impact COVID-19 had on psychological distress. In a study conducted on hemodialysis patients in 2010, 1417 patients completed PHQ-9 assessment and 238 (17%) patients showed moderate to severe depression [25]. In comparison, in this study, moderate to severe depression was observed in a relatively large proportion (about 23%) of dialysis patients and about 18% of all CKD patients, regardless of stage. It is difficult to make a simple comparison with previous studies. Thus, follow-up studies are needed after the COVID-19 pandemic has passed. Second, scales used in this study were self-reported measures. PHQ-9, GAD-7, IES-R, and ISI might fail to fully reflect depression, anxiety, psychological impact, and insomnia in CKD patients. Constructed questions might not reflect all COVID-19-related concerns, although we tried to overcome these limitations by using reliable and validated instruments widely used in other studies [8]. COVID-19-related concerns were constructed with reference to a prior research study conducted in China [8].

## 5. Conclusions

Although this study showed that anxiety and insomnia increased with increasing CKD stage, whether this had a direct correlation with COVID-19 is unclear. However, previous studies reported that patients with psychological problems, such as anxiety and insomnia, are vulnerable to COVID-19-related stress [9,26]. One study suggested that depression and anxiety can be associated with suicide, fatigue, sleep disorder, and pain in CKD patients with hemodialysis [27]. Therefore, as CKD stage increases, more attention should be paid to not only physical care, but also mental care for these patients.

## Figures and Tables

**Table 1:** Baseline characteristics of study subjects.

Variable	Total (*n* = 397)	CKD S1~S2 (*n* = 111)	CKD S3~S5ND (*n* = 138)	CKD S5D (*n* = 148)	*p*-Value
Characteristics					
Age (years)	55.2 ± 13.5	51.2 ± 10.9	60.3 ±14.9	53.5 ± 12.3	0.199
Sex–Male	249(62.25%)	72 (64.86%)	87 (63.04%)	88 (59.5%)	0.366
Visit interval (day)	68.9 ± 62.7	127.3 ± 57.4	79.0 ± 49.1	15.0 ± 15.8	<0.001
DM	141 (35.25%)	23 (20.72%)	63 (45.65%)	55 (37.26%)	0.014
HTN	304 (76.57%)	65 (58.56%)	115 (83.33%)	124 (83.78%)	<0.001
Statin	204 (51.0%)	55 (49.6%)	89 (64.5%)	60 (40.5%)	0.080
Laboratory data					
Hemoglobin (g/dL)	12.03 ± 2.77	14.07 ± 1.48	11.87 ± 2.10	10.61 ± 13.14	<0.001
Hematocrit (%)	35.59 ± 6.47	41.63 ± 4.26	35.50 ± 5.87	31.06 ± 4.37	<0.001
Blood urea nitrogen (mg/dL)	36.60 ± 21.52	15.09 ± 4.20	39.17 ± 32.46	52.83 ± 16.30	<0.001
Creatinine (mg/dL)	4.99 ± 4.78	0.92 ± 0.22	2.66 ±1.91	10.30 ± 3.50	<0.001
eGFR (ml/min)	37.85 ± 35.62	87.27 ± 16.69	31.86 ± 5.80	5.32 ± 2.48	<0.001
Potassium (mmol/L)	4.51 ± 0.58	4.33 ± 0.35	4.62 ± 0.55	4.54 ± 0.71	<0.001
Albumin (g/dL)	4.20 ± 0.46	4.49 ± 0.40	4.24 ± 0.42	3.94 ± 0.40	<0.001
Cholesterol (mg/dL)	159.36 ± 44.93	177.65 ± 47.29	159.55 ± 47.40	145.01± 34.78	0.087

CKD S5D, chronic kidney disease stage 5 dialysis; S3~S5ND, stage 3~stage 5 non-dialysis; S1~S2, stage 1~stage 2; DM, diabetes mellitus; HTN, hypertension; eGFR, estimated glomerular filtration rate.

**Table 2:** Associations between psychological distress and baseline characteristics of study subjects.

	OR (95% CI)	*p*-Value
Depression (PHQ-9 score 5~21)		
CKD		0.353
CKD S3~S5ND	1.451 (0.834~2.525)	0.188
CKD S5D	1.411 (0.828~2.405)	0.206
Sex (Male)	0.792 (0.519~1.210)	0.281
Age	1.064 (0.915~1.238)	0.418
DM	0.824 (0.528~1.284)	0.392
HTN	0.853 (0.516~1.409)	0.534
Moderate to severe anxiety (GAD-7 score 10~21) *		
CKD		0.013
CKD S3~S5ND	2.119 (1.152~3.898)	0.016
CKD S5D	2.341 (1.305~4.201)	0.004
Sex (Male)	0.607 (0.390~0.944)	0.027
Age	1.033 (0.882~1.211)	0.686
DM	0.785 (0.490~1.257)	0.314
HTN	0.598 (0.354~1.008)	0.054
Psychological impact of event (IES-R score 9~88)		
CKD		0.561
CKD S3~S5ND	0.741 (0.428~1.281)	0.283
CKD S5D	0.839 (0.494~1.423)	0.515
Sex (Male)	0.936 (0.612~1.432)	0.762
Age	1.019 (0.877~1.184)	0.811
DM	0.993 (0.640~1.539)	0.973
HTN	0.713 (0.428~1.188)	0.713
Insomnia (ISI score 8~28)		
CKD		<0.001
CKD S3~S5ND	2.535 (1.410~4.560)	0.002
CKD S5D	3.019 (1.715~5.314)	<0.001
Sex (Male)	0.690 (0.449~1.061)	0.091
Age	0.998 (0.857~1.163)	0.982
DM	0.984 (0.628~1.540)	0.943
HTN	0.693 (0.415~1.160)	0.163

Outcomes are simulated from a logistic regression model; OR, odds ratio; CI, confidence interval; CKD S3~S5ND, chronic kidney disease stage 3~stage 5 non-dialysis; S5D, stage 5 dialysis; PHQ-9, the 9-item Patient Health Questionnaire; GAD-7, the 7-item Generalized Anxiety Disorder scale; IES-R, the 22-item impact of Event Scale-Revised; ISI, insomnia severity index; DM, diabetes mellitus; HTN, hypertension. * Since the number of normal patients on the GAD-7 score was too small (*n* = 1), patients with moderate to severe anxiety were used as outcome to apply logistic regression.

**Table 3:** Comparison of score and severity of questionnaires covering mental health status between CKD stage 1~2 and CKD stage 3~5 with or without dialysis.

	CKD S1~S2 (*n* = 111)	CKD S3~S5D (*n* = 286)	*p*-Value
**PHQ-9**	4.01 ± 4.23	5.40 ± 5.99	0.026
Severity			0.028
Normal (0–8 points)	71 (64.0%)	164 (57.3%)	
Mild (9–14 points)	28 (25.2%)	63 (22.0%)	
Moderate (15–19 points)	8 (7.2%)	28 (9.8%)	
Severe (20–27 points)	4 (3.6%)	31 (10.8%)	
**GAD-7**	9.00 ± 3.69	10.37 ± 5.03	0.010
Severity			0.010
Normal (0–4 points)	0 (0%)	1 (0.3%)	
Mild (5–9 points)	84 (74.8%)	178 (62.2%)	
Moderate (10–14 points)	21 (18.9%)	64 (22.4%)	
Severe (15–21 points)	7 (6.3%)	43 (15.0%)	
**IES-R**	13.82 ± 10.31	15.63 ± 14.56	0.232
Severity			0.235
Normal (0–8 points)	41 (36.9%)	123 (43.0%)	
Mild (9–25 points)	57 (51.4%)	95 (33.2%)	
Moderate (26–43 points)	12 (10.8%)	50 (17.5%)	
Severe (44–88 points)	1 (0.9%)	18 (6.3%)	
**ISI**	5.62 ± 4.58	7.45 ± 5.61	0.002
Severity			<0.001
Normal (0–7 points)	81 (73.6%)	153 (53.5%)	
Mild (8–14 points)	29 (26.4%)	112 (39.2%)	
Moderate (15–21 points)	0 (0%)	19 (4.8%)	
Severe (22–28 points)	0 (0%)	2 (0.7%)	

CKD S3~S5D, chronic kidney disease stage 3~stage 5 dialysis; S1~S2, stage 1~stage 2; PHQ-9, the 9-item Patient Health Questionnaire; GAD-7, the 7-item Generalized Anxiety Disorder scale; IES-R, the 22-item impact of Event Scale-Revised; ISI, insomnia severity index.

**Table 4:** Comparison of concerns and precautionary measures for 2019 coronavirus disease (COVID-19) in CKD patients.

N (%) or Mean ± SD	CKD S1~S2 (*n* = 111)	CKD S3~S5ND (*n* = 138)	CKD S5D (*n* = 148)	*p*-Value
Likelihood of contracting COVID-19 during the current outbreak				
Very likely	2 (1.8)	2 (1.4)	1 (0.7)	0.195
Somewhat likely	2 (1.8)	2 (1.4)	4 (2.7)	
Not very likely	36 (32.4)	37 (26.8)	63 (42.6)	
Not likely at all	71 (64.0)	97 (70.3)	80 (54.1)	
Level of confidence in patient’s own doctor’s ability to diagnose or recognize				
Very confident	44 (39.6)	64 (46.4)	50 (33.8)	0.730
Somewhat confident	54 (48.6)	62 (44.9)	91 (61.5)	
Not very confident	6 (5.4)	4 (2.9)	2 (1.4)	
Not at all confident	7 (6.3)	8 (5.8)	5 (3.4)	
Likelihood of surviving if infected with COVID-19				
Very likely	14 (12.7)	22 (15.9)	22 (14.9)	0.560
Somewhat likely	42 (38.2)	59 (42.8)	58 (39.2)	
Not very likely	43 (39.1)	47 (34.1)	56 (37.8)	
Not likely at all	11 (10.0)	10 (7.2)	12 (8.1)	
Concerns about other family members getting COVID-19 infections				
Very likely	31 (27.9)	50 (36.5)	49 (33.1)	0.492
Somewhat likely	65 (58.6)	67 (48.9)	80 (54.1)	
Not very likely	10 (9.0)	14 (10.2)	14 (9.5)	
Not likely at all	5 (4.5)	6 (4.4)	5 (3.4)	
Covering mouth when coughing and sneezing				
Very likely	48 (43.2)	57 (41.3)	55 (37.2)	0.191
Somewhat likely	59 (53.2)	74 (53.6)	81 (54.7)	
Not very likely	2 (1.8)	5 (3.6)	10 (6.8)	
Not likely at all	2 (1.8)	2 (1.8)	2 (1.4)	
Avoiding sharing utensils (e.g., chopsticks) during meals				
Very likely	23 (20.7)	35 (25.4)	29 (19.6)	0.886
Somewhat likely	52 (46.8)	73 (52.9)	77 (52.0)	
Not very likely	31 (27.9)	22 (15.9)	35 (23.6)	
Not likely at all	5 (4.5)	8 (5.8)	7 (4.7)	
Washing hands immediately after coughing, rubbing the nose, or sneezing				
Very likely	22 (19.8)	25 (18.1)	30 (20.3)	0.941
Somewhat likely	54 (48.6)	70 (50.7)	71 (48.0)	
Not very likely	34 (30.6)	40 (29.0)	44 (29.7)	
Not likely at all	1 (0.9)	3 (2.2)	3 (2.0)	
Wearing mask regardless of the presence or absence of symptoms				
Very likely	69 (62.2)	57 (42.3)	81 (54.7)	0.968
Somewhat likely	34 (30.6)	71 (51.8)	61 (41.2)	
Not very likely	6 (5.4)	4 (2.9)	5 (3.4)	
Not likely at all	2 (1.8)	4 (2.9)	0 (0)	
Feeling that too much unnecessary worry surrounds the COVID-19 outbreak				
Very likely	12 (10.8)	19 (13.8)	22 (14.9)	0.138
Somewhat likely	43 (38.7)	51 (37.0)	62 (41.9)	
Not very likely	48 (43.2)	59 (42.8)	58 (39.2)	
Not likely at all	8 (7.2)	9 (6.5)	6 (4.1)	
Average number of hours stayed at home per day to avoid COVID-19				
Hours	13.00 ± 5.52	14.18 ± 6.63	15.92 ± 6.23	<0.001

CKD S5D, chronic kidney disease stage 5 dialysis; S3~S5ND, stage 3~stage 5 non-dialysis; S1~S2, stage 1~stage 2.

**Table 5:** Comparison of concerns and precautionary measures for 2019 coronavirus disease (COVID-19) between CKD stage 1~2 and CKD stage 3~5 patients with or without dialysis.

N (%) or Mean ± SD	CKD S1~S2 (*n* = 111)	CKD S3~S5D (*n* = 286)	*p*-Value
Likelihood of contracting COVID-19 during the current outbreak			
Very likely	2 (1.8)	3 (1.0)	0.897
Somewhat likely	2 (1.8)	2 (1.8)	
Not very likely	36 (32.4)	100 (35.0)	
Not likely at all	71 (64.0)	177 (61.9)	
Level of confidence in patient’s own doctor’s ability to diagnose or recognize			
Very confident	44 (39.6)	144 (39.9)	0.399
Somewhat confident	54 (48.6)	153 (53.5)	
Not very confident	6 (5.4)	6 (2.1)	
Not at all confident	7 (6.3)	13 (4.5)	
Likelihood of surviving if infected with COVID-19			
Very likely	14 (12.7)	44 (15.4)	0.270
Somewhat likely	42 (38.2)	117 (40.9)	
Not very likely	43 (39.1)	103 (36.0)	
Not likely at all	11 (10.0)	22 (7.7)	
Concerns about other family members getting COVID-19 infections			
Very likely	31 (27.9)	99 (32.9)	0.367
Somewhat likely	65 (58.6)	147 (51.6)	
Not very likely	10 (9.0)	28 (9.8)	
Not likely at all	5 (4.5)	11 (3.9)	
Covering mouth when coughing and sneezing			
Very likely	48 (43.2)	112 (39.2)	0.341
Somewhat likely	59 (53.2)	155 (54.2)	
Not very likely	2 (1.8)	15 (5.2)	
Not likely at all	2 (1.8)	4 (1.4)	
Avoiding sharing utensils (e.g., chopsticks) during meals			
Very likely	23 (20.7)	64 (22.4)	0.358
Somewhat likely	52 (46.8)	150 (52.4)	
Not very likely	31 (27.9)	57 (19.9)	
Not likely at all	5 (4.5)	15 (5.2)	
Washing hands immediately after coughing, rubbing the nose, or sneezing			
Very likely	22 (19.8)	55 (19.2)	0.835
Somewhat likely	54 (48.6)	141 (49.3)	
Not very likely	34 (30.6)	84 (29.4)	
Not likely at all	1 (0.9)	6 (2.1)	
Wearing mask regardless of the presence or absence of symptoms			
Very likely	69 (62.2)	139 (49.0)	0.176
Somewhat likely	34 (30.6)	132 (46.5)	
Not very likely	6 (5.4)	9 (3.2)	
Not likely at all	2 (1.8)	4 (1.4)	
Feeling that too much unnecessary worry surrounds the COVID-19 outbreak			
Very likely	12 (10.8)	41 (14.3)	0.268
Somewhat likely	43 (38.7)	113 (39.5)	
Not very likely	48 (43.2)	117 (40.9)	
Not likely at all	8 (7.2)	15 (5.2)	
Average number of hours stayed at home per day to avoid COVID-19			
Hours	13.00 ± 5.52	15.92 ± 6.23	0.002

CKD S3~S5D, chronic kidney disease stage 3~stage 5 dialysis; S1~S2, stage 1~stage 2.

## Data Availability

The authors confirm that the data supporting the findings are available from the corresponding author upon reasonable request.

## Supplementary Materials

The following supporting information can be downloaded at: www.mdpi.com/article/10.3390/jcm11164776/s1, Figure S1: Hours stayed at home to avoid COVID-19 and PHQ-9, GAD-7, IES, and ISI scores of patients were compared through correlation analysis. All showed positive correlations, although their correlation coefficients were not large. Only the correlation between PHQ-9 suggesting depression and time stayed at home showed a statistical significance. PHQ-9, the 9-item Patient Health Questionnaire; GAD-7, the 7-item Generalized Anxiety Disorder scale; IES-R, the 22-item impact of Event Scale-Revised; ISI, insomnia severity index; Table S1: Comparison of score and severity of questionnaires covering mental health status between non-dialytic CKD and dialysis CKD patients; Table S2: Comparison of concerns and precautionary measures for 2019 coronavirus disease (COVID-19) between non-dialytic CKD and dialysis CKD patients.
