# Supplementary material for: The Higher the CKD Stage, the Higher the Psychological Stress in Patients with CKD during COVID-19 Pandemic
Source: J Clin Med. 2022 Aug 16;11(16):4776. doi: 10.3390/jcm11164776 (PMC9409632; doi:10.3390/jcm11164776)
Supplement: Supplementary file 1 [file jcm-11-04776-s001.zip › jcm-1811869-supplementary.pdf]

Supplementary Table S1. Comparison of score and severity of questionnaires covering mental health status between non-dialytic CKD and dialysis CKD patients

	**Non-dialytic CKD (*n* = 249)**	**Dialysis (*n* = 148)**	***p* - value**
**PHQ-9**	4.67±5.06	5.57±6.36	0.121
Severity			0.084
Normal (0-8 points)	146 (59.8%)	85 (57.4%)	
Mild (9-14 points)	64 (25.7%)	28 (18.9%)	
Moderate (15-19 points)	20 (8.0%)	16 (10.8%)	
Severe (20-27 points)	16 (6.4%)	19 (12.8%)	
**GAD-7**	9.67±4.42	10.51±5.19	0.086
Severity			0.096
Normal (0-4 points)	0 (0%)	1 (0.7%)	
Mild (5-9 points)	171 (68.7%)	90 (60.8%)	
Moderate (10-14 points)	52 (20.9%)	33 (22.3%)	
Severe (15-21 points)	26 (10.4%)	24 (16.2%)	
**IES-R**	15.16±13.24	15.07±14.01	0.949
Severity			0.479
Normal (0-8 points)	102 (41.0%)	62 (41.9%)	
Mild (9-25 points)	102 (41.0%)	50 (33.8%)	
Moderate (26-43 points)	34 (13.7%)	28 (18.9%)	
Severe (44-88 points)	11 (4.4%)	8 (5.4%)	
**ISI**	6.39±5.11	7.88±5.74	0.008
Severity			<0.001
Normal (0-7 points)	158 (63.7%)	76 (51.4%)	
Mild (8-14 points)	89 (35.9%)	52 (35.1%)	
Moderate (15-21 points)	1 (0.4%)	18 (12.2%)	
Severe (22-28 points)	0 (0%)	2 (1.4%)	

CKD, chronic kidney disease; PHQ-9, the 9-item Patient Health Questionnaire; GAD-7, the 7-item Generalized Anxiety Disorder scale; IES-R, the 22-item impact of Event Scale-Revised; ISI, insomnia severity index.

Supplementary Table S2. Comparison of concerns and precautionary measures for 2019 coronavirus disease (COVID-19) between non-dialytic CKD and dialysis CKD patients

N (%) or mean ± SD	Non-dialysis CKD (*n* = 249)	Dialysis (*n* = 148)	*p* - value
Likelihood of contracting COVID-19 during the current outbreak			
Very likely	4 (1.6)	1 (0.7)	0.042
Somewhat likely	4 (1.6)	4 (2.7)	
Not very likely	73 (29.3)	63 (42.6)	
Not likely at all	168 (67.5)	80 (54.1)	
Level of confidence in patient's own doctor's ability to diagnose or recognize			
Very confident	108 (43.4)	50 (33.8)	0.833
Somewhat confident	116 (46.6)	91 (61.5)	
Not very confident	10 (4.0)	2 (1.4)	
Not at all confident	15 (6.0)	5 (3.4)	
Likelihood of surviving if infected with COVID-19			
Very likely	36 (14.5)	22 (14.9)	0.956
Somewhat likely	101 (40.7)	58 (39.2)	
Not very likely	90 (36.3)	56 (37.8)	
Not likely at all	21 (8.5)	12 (8.1)	
Concerns about other family members getting COVID-19 infections			
Very likely	81 (32.7)	49 (33.1)	0.762
Somewhat likely	132 (53.2)	80 (54.1)	
Not very likely	24 (9.7)	14 (9.5)	
Not likely at all	11 (4.4)	5 (3.4)	
Covering mouth when coughing and sneezing			
Very likely	105 (422)	55 (37.2)	0.198
Somewhat likely	133 (53.4)	81 (54.7)	
Not very likely	7 (2.8)	10 (6.8)	
Not likely at all	4 (1.6)	2 (1.4)	
Avoiding sharing utensils (e.g., chopsticks) during meals			
Very likely	58 (23.3)	29 (19.6)	0.539
Somewhat likely	125 (50.2)	77 (52.0)	
Not very likely	53 (21.3)	35 (23.6)	
Not likely at all	13 (5.2)	7 (4.7)	
Washing hands immediately after coughing, rubbing the nose, or sneezing			
Very likely	47 (18.9)	30 (20.3)	0.943

Somewhat likely	124 (49.8)	71 (48.0)	
Not very likely	74 (29.7)	44 (29.7)	
Not likely at all	4 (1.6)	3 (2.0)	
Wearing mask regardless of the presence or absence of symptoms			
Very likely	127 (51.2)	81 (54.7)	0.185
Somewhat likely	105 (42.3)	61 (41.2)	
Not very likely	10 (4.0)	5 (3.4)	
Not likely at all	6 (2.4)	0 (0)	
Feeling that too much unnecessary worry surrounds the COVID-19 outbreak			
Very likely	32 (12.4)	22 (14.9)	0.152
Somewhat likely	94 (37.8)	62 (41.9)	
Not very likely	107 (43.0)	58 (39.2)	
Not likely at all	17 (6.8)	6 (4.1)	
Average number of hours stayed at home per day to avoid COVID-19			
Hours	13.65 ± 6.17	15.92 ± 6.23	< 0.001

CKD, chronic kidney disease.

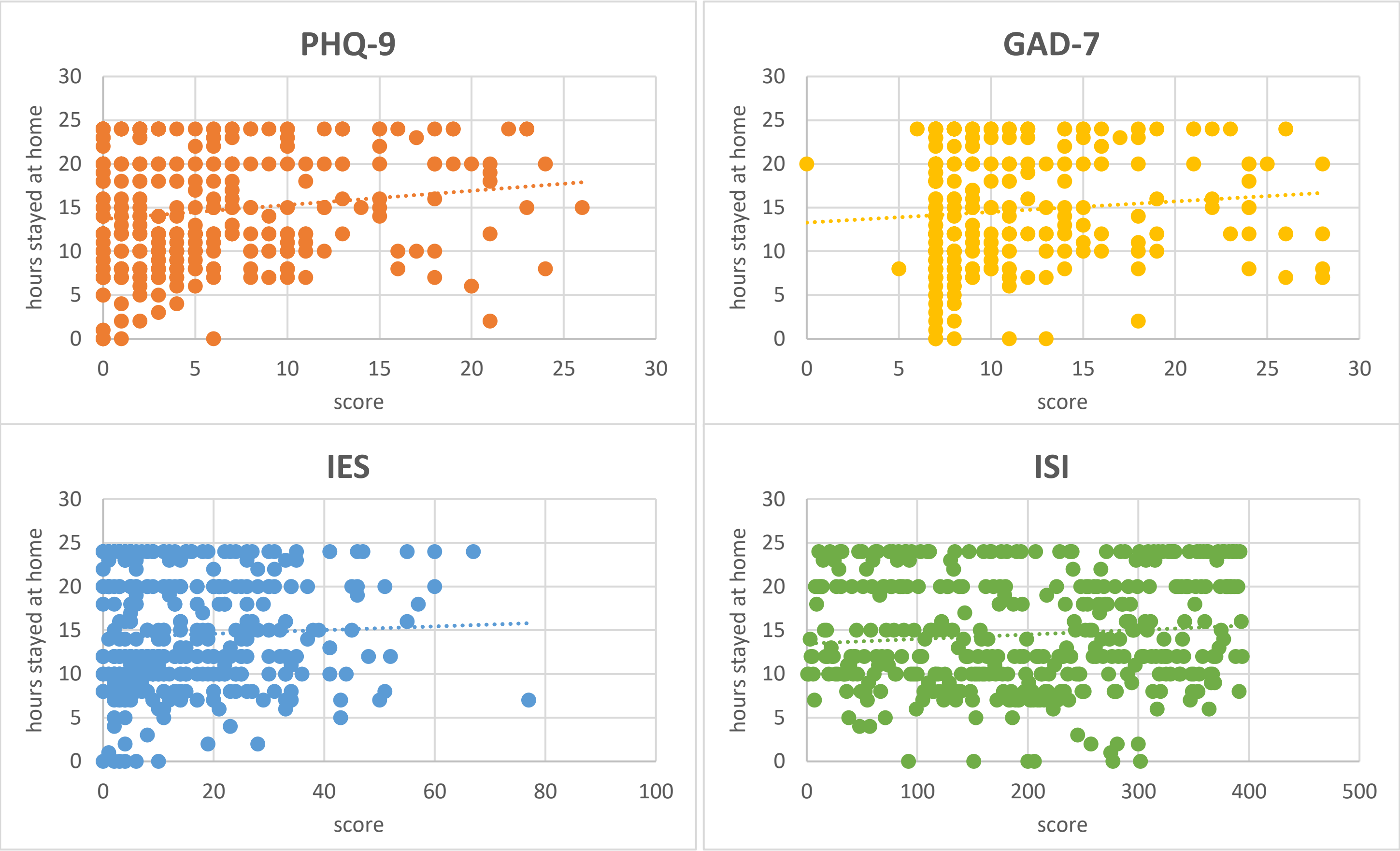

	PHQ-9	GAD-7	IES	ISI
Correlation coefficient	0.144665	0.090973	0.045209	0.073286
Coefficient determination	0.020928	0.008276	0.002044	0.005371
p-value	0.004008	0.071267	0.370797	0.146496

Supplementary Figure S1. Hours stayed at home to avoid COVID-19 and PHQ-9, GAD-7, IES, and ISI scores of patients were compared through correlation analysis. All showed positive correlations, although their correlation coefficients were not large. Only the correlation between PHQ-9 suggesting depression and time stayed at home showed a statistical significance. PHQ-9, the 9-item Patient Health Questionnaire; GAD-7, the 7-item Generalized Anxiety Disorder scale; IES-R, the 22-item impact of Event Scale-Revised; ISI, insomnia severity index.
